# Evolution of Iran’s health research system over the past 50 years: a narrative review

**DOI:** 10.7189/jogh.08.020703

**Published:** 2018-12

**Authors:** Parisa Mansoori

**Affiliations:** Centre for Global Health Research, Usher Institute of Population Health Sciences and Informatics, University of Edinburgh, Edinburgh, UK

## Abstract

**Background:**

A substantial growth has been reported in Iran’s health research output over the last recent decades, throughout the times of economic, social, and political instability. This study reviewed the existing literature to provide a better understanding of the evolution of Iran’s health research system over this period.

**Methods:**

A narrative review of studies addressing health research system (HRS) in Iran was performed. The search strategy and categorization of the retrieved data was informed by the HRS framework of the World Health Organization (WHO). This framework proposes four functions for HRS: (i) stewardship; (ii) financing; (iii) creating and sustaining resources; and (iv) producing and using research. Searches in MEDLINE through PubMed (using MeSH terms) complemented with semantic searches through PubMed and Google Scholar were conducted.

**Results:**

After removing the duplicates, 805 articles were retrieved, of which 601 were irrelevant, and 204 were reviewed.

**Conclusions:**

Iran has made substantial progress in different components of its HRS over the last few decades, such as starting a discourse surrounding health research ethics, priority-setting, and placing monitoring mechanisms while increasing the capacity for conducting and publishing research. However, there is still room for improvements, or even a need for fundamental changes, in several components, such as regarding increasing the research budget and improving the funding allocation mechanisms; improving the education curriculum; and promoting the use of evidence. The findings emphasized that improvement of HRS functions requires addressing context-specific problems. This review provides essential lessons to share with other low- and middle-income countries and international organizations, eg, the WHO.

Health research is increasingly regarded as an essential tool both for improving population health and for development [1-3]. Hence, numerous efforts at the national, regional, and global levels have attempted to strengthen health research capacity in low- and middle-income countries (LMICs) [2,4,5]. However, most LMICs continue to have limited capacity for health research [2,6,7]. Many have remained largely dependent on international academic institutions and donors: on the former for research-relevant knowledge and expertise, and on the latter for financial resources [2,6,8]. Evidence shows that many of the barriers impeding improvement of health research are shared across LMICs [2]. Thus, studying the approaches that each country takes for overcoming some common obstacles could provide important lessons to other LMICs.

Iran is a middle-income country which studying the changes in its health research could lead to valuable lessons for exchange. A substantial growth in Iran’s health research output has occurred over the last few decades [9], throughout the times of social, political, and economic instability, including almost 40 years of international sanctions [10]. An increase has been reported both in the quantity and in the citation impact of Iranian publications that are indexed in international bibliographic databases [9,11-14]. For instance, the number of Iranian annual publications in MEDLINE had increased from only 273 in 2000 to 14 511 in 2014 [15]. Likewise, Iran's global ranking based on citation counts in Scopus database was reported as having had improved from 56^th^ to 22^nd^ between 1996 and 2014 [16]. There has clearly been a rather significant evolution in the development of Iran’s capacity for health research. There is a need to explore and explain the various stakeholders, institutions, policies, the structures, and incentives in the academic community, and funding sources that underlie the observed increase in Iran’s health research output. This paper aims to review the existing literature that could provide a better understanding of the evolution of Iran’s HRS over the period of the past five decades.

Taking a systems perspective is recommended in studying health research performance at the national level [17]. A systems perspective to health research recognizes that a comprehensive, integrated, and coordinated approach is needed to understand and guide the production and the utilization of research to improve health outcomes and to decrease health inequities [2,17]. This approach was initially mentioned in the 1990 report of the Commission on Health Research for Development, stating that: “*research is a system involving people, institutions and processes*” [1]. In 2003, a conceptual framework for operationalizing a systems perspective to health research was published in the Bulletin of the World Health Organization (WHO) [17], which was further explained in the WHO 2004 World Report on Knowledge for Better Health [18]. The framework proposed four main functions for HRS including: (i) stewardship; (ii) financing; (iii) creating and sustaining resources; and (iv) producing and using research[17]. The review presented here will use this established framework to explore and discuss the key factors and events that have contributed to the evolution of Iran’s HRS.

## METHODS

Due to the broad scope of the topic and anticipated heterogeneity of the studies to review, a narrative review approach was employed, as it provides a rather comprehensive coverage [19,20]. A narrative review of the publications addressing health research system (HRS) in Iran was conducted. The search strategy and categorization of the retrieved data were informed by the HRS framework introduced by the WHO [17]. The framework proposes four functions for HRS. Components of each function are summarized in **Table 1**.

**Table 1:** Summary of the functions and components of the WHO health research system framework*

Function	Operational component
Stewardship	• Define and articulate vision for a national health research system (HRS)
	• Identify appropriate health research priorities and coordinate adherence to them
	• Set and monitor ethical standards for health research and research partnerships
	• Monitor and evaluate the HRS
Financing	• Secure research funds and allocate them accountably
Creating and sustaining resources	• Build, strengthen, and sustain the human and physical capacity to conduct, absorb, and utilize health research
Producing and using research	• Produce scientifically valid research outputs
	• Translate and communicate research to inform health policy, strategies, practices, and public opinion
	• Promote the use of research to develop new tools (drugs, vaccines, devices, and other applications) to improve health

*Table adapted from “Knowledge for better health: a conceptual framework and foundation for health research systems”, by Pang T et al [17].

The search was conducted in April 2018 through PubMed and Google Scholar. PubMed was used to search MEDLINE; relevant MeSH terms (ie, Medical Subject Headings) in conjunction with “Iran” in the Title and/or Abstract were used. Since HRS is a broad topic, semantic searchers – both through PubMed and Google Scholar – were also conducted to retrieve further relevant publications, either from journals or the gray literature. In Google Scholar, no difference was found in the number of search results for “Iran” and ‘Iran’. Hence only one variant was used; the term “Iran” was used in conjunction with relevant terms based on the four functions of the WHO HRS framework. In PubMed, all the search results were included. In Google Scholar, the results were sorted by relevance, and the inclusion of the search results was continued until it became clear that the listed results were no longer relevant. Full details of the search strategy are provided in **Box 1**.

Box 1 **Search strategy for the review of Iran’s health research system**

Search through PubMed:

1. (“Bibliometrics”[Mesh]) AND iran[Title/Abstract]
2. (“Ethics, Research”[Mesh]) AND iran[Title/Abstract]
3. (“Research”[Mesh]) AND iran[Title/Abstract]
4. (“Scientific Misconduct”[Mesh]) AND iran[Affiliation]
5. (“Periodicals as Topic”[Mesh]) AND iran[Title/Abstract]
6. (“Translational Medical Research”[Mesh]) AND iran[Title/Abstract]
7. ((“research system” OR “research policy”)) AND iran[Title/Abstract]
8. (“research capacity”) AND iran[Title/Abstract]
9. ((“research output” OR “research product” OR “research growth” OR “scientific growth” OR “scientific output” OR “scientific product”)) AND iran[Title/Abstract]
10. ((“research evaluation” OR “research assessment” OR “academic assessment” OR “academic evaluation”)) AND iran[Title/Abstract]
11. ((“research quantity” OR “research quality” OR “research impact”)) AND iran[Title/Abstract]
12. ((“scientometrics” OR “scientometric” OR “bibliometrics” OR “bibliometric”)) AND iran[Title/Abstract]
13. ((“research network”[Title/Abstract] OR “research networks”[Title/Abstract])) AND Iran[Affiliation]
14. (knowledge transfer[Title/Abstract]) AND iran[Affiliation]
15. “plagiarism” AND “Iran”

Search through Google Scholar:

1. “iran” AND “medical research”

Up to 50 pages

1. “iran” AND “health research”

Up to 15 pages

1. “iran” AND “research assessment”

Up to 10 pages

1. “iran” AND “research evaluation”

Up to 10 pages

1. “iran” AND “research priority”

Up to 12 pages

1. “iran” AND “research capacity” AND “medical”

Up to 11 pages

After removing the duplicates, 805 sources of information remained. Among them, 536 were found to be irrelevant after scanning the titles and abstracts and they were excluded. Full-text versions of the remaining 269 records were scanned. In the end, 204 records were retained as relevant to this narrative review, and they were reviewed in detail. All types of documents with available full-texts (either in English or Persian) were included.

The included 204 records were organized within Endnote (a reference management software) into the following categories: (i) financial, human, and infrastructural resources; (ii) knowledge networks and collaboration; (iii) medical/research/publication ethics; (iv) HRS monitoring and evaluation; (v) research priority-setting; (vi) national vision and agendas for health research; (vii) research output (general); (viii) bibliometric analyses; (ix) quality of publications; (x) disseminating and using research; and (xi) Iranian journals. This categorization was done following the initial familiarization with the topics covered by each record while guided by the HRS framework. After further familiarization with the data, the reviewed information were categorized as described in **Box 2**.

Box 2 **The categorization of the retrieved data on Iran’s health research system**

Stewardship:

- Structure and vision for governing health research
- Identification of health research priorities and coordinating adherence to them
- National-level ethical oversight
- Monitoring and evaluating HRS

Financing:

- Gross Domestic Expenditure on Research and Development (GERD)
- % of Research and Development (R&D) allocated to health
- % of health budget allocated to research
- Source of the research budget
- Mechanisms for distributing funds
- Mechanisms for tracking the investments

Creating and sustaining resources:

- Figures for human and infrastructural resources
- Capacity building – outcome and the barriers to research activities
- Collaboration

Producing, disseminating, using research:

- Number of research outputs, eg, research papers, books, patents
- Figures for Iranian journals
- Figures for citation-based indicators
- Underlying reasons for the growth of Iranian research publications
- Knowledge translation and research impact

## RESULTS AND DISCUSSION

Information about different components of Iran’s HRS since 1970 was extracted from the 204 reviewed documents which is presented and discussed in this section.

### Stewardship

#### Structure and vision for governing health research

Several organizations in Iran are actively involved with health research policy-making, either directly or indirectly [21]: (i) The Islamic Consultative Assembly (The Parliament); (ii) The Supreme Council for Cultural Revolution (the function of the Council is to define general policies in the field of higher education and culture); (iii) The Department of Research and Technology of Ministry of Health and Medical Education (MOHME); and (iv) The Plan and Budget Organization (ie, responsible for compilation of the annual budget) [21]. The Department of Research and Technology of MOHME, and (to a lesser extent) the research departments of the medical universities all across Iran, are responsible for the executive planning of the policies to achieve the national health research policies [21].

A national evaluation of different functions of HRS in Iran, which was published in 2004 [21], indicated that although an elaborate system existed in Iran to undertake the different operational components of the function ‘stewardship’, a clear articulation of the vision and the goals of health research was absent [21]. Later on, in 2009, a panel of Iranian experts drafted a “national scientific plan” for health, which outlined Iran’s long-term plan by 2025 [22]. The plan was based on the Islamic-Iranian values, an agenda known as Iran’s Vision for the year 2025, and the general concepts of the National Innovation System [22]. This national scientific plan outlined Iran’s national vision, goals, monitoring and evaluation indicators, priorities, policies, and strategies for health research [22].

No information was found in the reviewed literature about the extent to which this national scientific plan for health had been followed. It was suggested, as a general conclusion, that perhaps too often the policies are not well implemented in Iran. This may be explained in view of the following constraints [21]: (i) inconsistency in policies; (ii) instability in administration; (iii) limited alignment of policies and available facilities; (iv) lack of communication between researchers and policy-makers; and (v) the absence of suitable implementers for the policies [21]. Regarding the latter, many of the people at executive roles who should implement the policies seem to lack the necessary skills to fulfil their responsibilities. For example, an assessment of individuals in research management positions at 39 Iranian universities of medical sciences found that 40% of them lacked adequate research management skills [23].

#### Identifying health research priorities and coordinating adherence to them

According to the literature [21], four national-level health research prioritization exercises have been conducted in Iran. The first two, had been undertaken by the then National Research Council, one exercise in 1993, and another one on three separate occasions in 1991, 1995, and 1999. The last two exercises, were carried out by the Department of Research and Technology of MOHME (in 1996 and 2010-2011) [21]. The first three exercises had followed the Council on Health Research for Development’s approach (COHRED) [21], as COHRED proposes a process for national-level exercises to show essential steps for priority-setting processes [24]. The last exercise had used the Essential National Health Research (ENHR) method and had involved all the universities of medical sciences affiliated with MOHME [25]: local research priorities were identified by the universities who had also engaged stakeholders within the process. In this last exercise, a total number of 9607 research ideas were gathered from the universities, which after excluding the irrelevant ones were reduced to a list of 6723 ideas as “research priorities”. The research ideas were categorized into nine main areas, eg, communicable and non-communicable diseases, basic sciences, and health systems research [25,26].

In terms of institutional-level priority-setting for health research, 45 Iranian medical universities and 53 research centers were surveyed for their status of health research priority-setting in 2002 [21]. Twenty-eight of the universities had conducted at least one exercise [21]. Those that had not carried out any had stated that their research priorities were the same as the ones identified by MOHME [21]. Of the 53 surveyed research centers, 21 had never conducted any priority-setting, and 8 had based priorities upon the health research system’s problems [21]. Fifteen out of 25 executive departments in the health system had conducted a priority-setting exercise; the remaining 10 had indicated that their priorities corresponded to those set by MOHME [21]. The prioritization exercises had followed COHRED guidelines with some modification [21]. Over the last 10 years, several priority-setting exercises had been undertaken in Iran in different areas of health research, and on different levels of national, institutional, or regional as summarized in Table S1 in **Online Supplementary Document(Online Supplementary Document)**.

In 2002, a study commissioned by the WHO [21] attempted to define how research priorities were being identified in Iranian medical universities, in their affiliated research centers, and executive departments. Moreover, it studied how different stakeholders contributed to the processes, what information sources were used, how consensus was achieved, and what criteria were applied to set priorities [21]. The study concluded that “qualitative and quantitative techniques have not been used in these settings [Iranian medical universities] and the criteria used were diffuse; the one most frequently used was feasibility” [21]. It has also been mentioned that the attempts for setting health research priorities in Iran have so far been mainly relying on the opinion of experts [23].

In a qualitative study that investigated the barriers to evidence-based decision-making in Iran’s health system in 2012, participants identified the following challenges: (i) absence of a systematic prioritization mechanism; (ii) priorities being set by MOHME and not being communicated with academics; and (iii) priorities being too general that fail to guide researchers [27]. Another paper had looked into research projects that were approved between 2005 and 2007 by an Iranian university (Golestan University of Medical Sciences) and had found that half of the researchers had chosen the topic of their studies according to their “personal interest” [28]. Finally, a bibliometric study mapped the number of research publications in Iran against the burden of disease in the country and found that the publications did not seem to be aligned with the disease burden [29].

The author could not find any study that had matched the investments in health research with research priorities. However, MOHME had stated in 2013 – drawing on the data collected for the annual evaluation of academic performance of medical universities in Iran – that 70% of research projects conducted at universities were in line with their institutional-level priorities [30]. It was also reported that the topic of 60% of the projects undertaken in 2007 in Iranian medical universities was aligned with their institutional priorities [31]. Nonetheless, adherence to the priorities would matter only once the priorities are identified through systematic, inclusive, and transparent processes.

Some of the barriers to initiation of health research prioritization exercises in Iran and to the utilization of their findings have been identified as follows: (i) the rapid turnover of people at executive roles, which makes individuals at such relatively temporary positions reluctant to engage with time-consuming processes [32]; (ii) poor knowledge of the majority of research directors of priority-setting methods [32]; (iii) the highly centralized and top-down decision-making process in Iran’s HRS [27,32]; (iv) inadequate interaction between academia and the end-users of health research [32]; (v) lack of incentives for the researchers who are invited to participate in priority-setting exercises [32];(vi) the identified priorities often being too general [27,32]; (vii) most decision-maker attending the meetings only as a formality, without providing any “intellectual input” [32]; and (viii) “powerful” individuals with louder voices influencing the opinion of others, such as the representatives from non-governmental organizations (NGOs), in consensus-based prioritization exercises [32].

#### National-level ethical oversight

In terms of introducing guidelines and regulations regarding medical and biomedical research ethics, Iran has made significant progress over the last 25 years (some major activities are summarized in **Box 3****)**. While until the mid-1990s, not much attention was directed towards ethical aspects of medical research in Iran, in the late 1990s, a paradigm shift seemed to have happened [41]; some reasons to explain this were suggested, and they will be presented in the further text.

Box 3 **Iran’s activities for promoting medical ethics (including medical and biomedical research ethics)**

**1993:** MOHME supported the establishment of a research center focused on medical ethics: Medical Ethics Research Center – MEHRC. In the same year, the center held the first international conference on medical ethics in Tehran, which saw the collection, organization, and categorization of a number of scientific publications on medical ethics, and the publication of proceedings of the conference [21,33]. Over the following years, MEHRC continued promoting medical ethics by holding numerous seminars and courses for health care professionals and academics [21,33]. The center has published several books, including a comprehensive textbook of medical ethics that is used in Iranian medical schools [21].

**1997-1998**: National Committee of Ethics in Medical Research was formed in MOHME to: (i) apply Islamic, legal, and moral principles to medical research; (ii) guard human rights in research and legally protect the participants, the researchers, and the institutions involved; and (iii) promote mandatory inclusion of advisors on ethical issues in all research projects at universities, private research foundations, and industries [34].

**1999:** Committees of Ethics in Medical Research were formed at the institutional level to monitor the alignment of research that is conducted at universities of medical sciences and biomedical research centers with the national and international ethics principles [21,26]. The committees were asked to follow a uniform guideline, which was written by the Department of Research at MOHME [35]. Also, research ethics courses have been held periodically to train the ethics committee members at the universities [36].

**2000:** MEHRC took the initiative to enact a code of medical research ethics [21]. Preparation of these codes was done through a 1.5-year project consisting 46 national ethics committee meetings at MOHME through: (i) a comprehensive review of international codes of ethics, eg, the Helsinki Declaration and documents of the Council for International Organizations of Medical Sciences (CIOMS); and (ii) customizing the international standards for the context of Iran [33,37]. To better understand the context of the Iranian society regarding research ethics, a national survey was conducted, and the relevant codes in religious laws in Shia (the official religion in Iran) were further reviewed [35,38]. Finally, a code of practice of 26 principles was developed for research that involves human subjects [21]. Examples of the codes were a requirement of informed consent; the need to review the risks and benefits of each study; protection of participants’ rights; confidentiality of participants’ information; compensation for injury; and preservation of the rights of the fetus, prisoners, and individuals with mental illnesses [38].

**2002:** The Department of Research and Technology of MOHME initiated compiling a strategic plan for medical ethics activities in Iran [33]. The “strategic plan” covered areas from management, regulations, education, and training to monitor and assessment of medical ethics activities at the national level [33]. The year 2002 was particularly crucial for biomedical research in Iran, because in that year, Iran’s Supreme Leader, Ayatollah Ali Khamenei, released a religious decree (fatwa) in which experiments using human embryonic stem (hES) cells were permitted under special circumstances [39]. Finally, because in the 26-item codes of medical research, the ethics codes for genetic studies were minimal, some Iranian researchers in 2002 initiated reviewing of the world literature about ethical standards for genetic studies to investigate how they could be adapted for the Iranian context [35].

**2005-2006:** After rigorous reviewing of relevant literature both on ethics and religious principles, Specific National Ethical Guidelines for Biomedical Research were drafted jointly by (i) MEHRC, (ii) Department of Research and Technology of MOHME, and (iii) the Endocrinology and Metabolism Research Center of TUMS [33]. The draft was revised by a group of experts in law, ethics, and medicine as well as the religious authorities [36]. Then, the revised guidelines were reviewed, approved, and ratified by the Iranian Parliament and the Guardian Council of the Constitution, and were delivered to all the medical universities and research centers [36,40]. The guideline included 22 items, addressing clinical trials; research involving vulnerable groups; genetic research; research on gamete and embryo; transplantation research; and research on animals [33]. It prohibited the production of human embryos for research purposes or production of human-animal hybrids, and eugenics [39].

Shortly after the release of the first revision of the Declaration of Helsinki (in 1975), Iran went into a turbulent decade: the Islamic Revolution took place in 1979, followed by the Iran-Iraq war (1980-1988) [41]. The urgent challenges that Iran was facing during the 1980s and the early 1990s did not allow implementation of the recommendations of the Helsinki Declaration [41]. Additionally, following the war in which Iraq seemed to be backed by the West against Iran [42], a period of hostility between Iran and the West began, which resulted in Iran’s isolation, even from the international scientific community [43]. It is suggested that this could be a reason that the Iranian academics who were trained during that period became rather unfamiliar with the international research standards [43]. This meant that, until the mid-1990s, not only had Iran lacked essential resources to move towards improving health research practices but also there may not have been sufficient interest in this progress. It is even argued that perhaps the long history of medical sciences in Iran, along with the stress placed on cultural values and religious beliefs in the country, had undervalued the need for a new set of ethical standards [21,38].

However, when Iran began to publish its research output in international journals, the need for aligning activities with international research standards became a lot more apparent [41]. Because for instance, international journals requested authors to provide information about the ethical considerations of their research upon submitting manuscripts for publication [41]. Furthermore, addressing ethical issues became particularly important in biomedical research, where some new areas, eg, stem cell research, were emerging in which Iran could potentially pioneer [41,44].

Finally, in the late 1990s, some leading medical researchers in Iran called for urgent action towards addressing ethical aspects of health research in Iran. In their 1999 paper [41], the need for attention to medical research ethics was highlighted by focusing on the poor status of ethics in clinical trials. The study [41] had assessed 51 clinical trials conducted between 1995 and 1998 in Tehran University of Medical Sciences (TUMS), ie, the leading Iranian medical university – presumably having a better performance than the rest. Only one trial had mentioned ‘ethical considerations’ in its proposal and/or final report; in only six of the 51, human subjects were informed that they were participating in a research [41]. Obtaining informed consent was mentioned in only four of the reports; 13 of the trials had used placebo while in 10 of them, the participants were imposed to some risks without having been informed that they might receive placebo. In more than 80% of the trials, the participants had even paid for the intervention, because they thought those were part of their treatment [41].

As evidence of the progress that has taken place since then, a survey of ethics committees of the Iranian medical universities reported that in 2011, all the universities had ethics committees (with 5 to 11 members each) [45]. 95% of the committees had a template consent form to provide to the researchers; all would have reviewed research that involved human participants; and in half of the universities, non-compliance with the regulations would have led to penalties [45]. Another study examined grant applications that had been approved during 2003-2008 at one university (Urmia University of Medical Sciences) [46]. Eighty percent of applications for conducting clinical trials had included informed consent. Of the total 324 applications (including all types of research), 85.5% had addressed ethical considerations [46].

It is of much interest to understand how the processes that have led to the compilation of guidelines in medical and biomedical research ethics in Iran had been facilitated. The literature primarily highlights the pivotal role of one person in this regard: Professor Bagher Larijani, one of the former chancellors of TUMS who also founded the Medical Ethics Research Center (MEHRC) in Iran [41]. It is argued that the reason why Professor Larijani was able to successfully lead discussions surrounding medical and biomedical research ethics – which is a potentially sensitive issue in most countries [35,47] – is that not only he is a prominent medical practitioner and leading researcher in Iran but also he came from a highly religious and politically influential family [41].His father was a religious authority and his brothers have always held important decision-making positions in the Islamic Republic of Iran [41]. Therefore, Larijani could use the confidence that Iranian authorities had in him as an opportunity [41] and he played a crucial role in (i) promoting the activities of MEHRC both among the policy-makers in the health system and among the Islamic scholars; and consequently, (ii) developing medical research ethics guidelines in Iran [40]. Another facilitator of the improvements was suggested to have been the support of the Iranian political leaders [36]. It was assumed that this support could have had partly originated in their interest in advancing Iran’s international rank based on science and technology indicators [40].

Regarding Iran’s advances in research ethics, the literature shows that not only Iran has significantly progressed in terms of the development of national medical and biomedical research ethics guidelines and has promoted their use through training and by introducing regulations but also research ethics of a diverse range of specific issues have been discussed by Iranian scholars over the past years. For example, ethical aspects of involving Iranian female participants [38]; ethical evaluation of research projects that are funded by international organizations [48]; ethical issues in clinical trials [49]; or research on laboratory animals [50,51] are all discussed elaborately in the literature.

Steps have also been taken towards promoting integrity in research publications. It was mentioned in 2012 that the number of Iranians who were members of the Committee on Publication Ethics (COPE), World Association of Medical Journal Editors (WAME), and/or European Association of Science Editors (EASE) had substantially increased [11]. A survey of 27 Iranian medical journal editors back in 2001 indicated that the majority of the editors had an average to high knowledge of the Uniform Requirements for Manuscripts Submitted to Biomedical Journals [52]. Moreover, it had been pointed out that in 2011 the first scientific congress of the Iranian Society of Medical Editors was held in Iran, in collaboration with COPE and with a focus on ‘publication ethics’ [53].

In addition to this, several Iranian journals have been trying to raise awareness among academics about research integrity and publication ethics in recent years [54-57]. For example, a paper depicted several examples of *good practice* (eg, obtaining permission before reproducing figures protected by copyright) and examples of *research and/or publishing misconduct* (eg, data fabrication, ghost and guest authorship) followed by relevant recommendations based on COPE guidelines [58]. The examples were from papers authored by the faculty members of a large Iranian university (Mashhad University of Medical Sciences), which had been published in the journals owned by the same university [58]. Finally, some universities, eg, Shiraz University of Medical Sciences, had not only been providing short courses on publication ethics but also had been offering an MS program on medical journalism since 2008 [59].

Despite such efforts, the literature still calls for greater attention towards research integrity and publication ethics in Iran. In 2008, the journal *Nature* reported several cases of retraction of publications by senior Iranian officials, albeit outside medical fields, due to apparent plagiarism [60]. A recent paper has identified the retracted documents from Open Access Journals in MEDLINE and has indicated that the majority of the retracted publications were authored by researchers affiliated with institutions in China (n = 199), India (n = 83), USA (n = 75), and Iran (n = 50) [61]. It was reported that in 2016, 28 retractions from Iranian authors were the result of compromising the peer review process, plagiarism, and authorship disputes [61]. An investigation of the prevalence of publication misconduct in the papers published in Iranian journals indexed in Scopus database during 2009-2011 reported guest authorship (18.10%) and falsification of the methodology (12.65%) as the most common types of misconduct [62]. Another study reported that nearly 26% of postgraduate students who graduated from one of the medical schools in Iran in 2015 had done some sort of research misconduct (including plagiarism, fabrication, or falsification of data) in their theses [63]. In 2009, an Iranian medical journal studied a sample of 80 of the manuscripts received by their journal and found that 55% of the manuscripts had at least one plagiarized sentence [56].

The findings of a survey in 2012 described the knowledge of medical students at TUMS (ie, the leading Iranian medical university) about plagiarism and self-plagiarism as very low [64]. It was reported in another survey that nearly 10% of students at TUMS were not even aware that using a copied paragraph from a textbook or a web page in their academic writing is unacceptable [65]. As for postgraduates, a recent study has analyzed the curricula of 125 postgraduate programs in medical sciences in Iran and has found that only 53 programs (42%) contained ethics training, of which only 17 had specific courses on research ethics, and even that was an elective subject in 25% of the programs [66]. It had been thought that medical ethics education had been improved by the launch of MPH (Master of Public Health) courses in 2004 as the program includes medical ethics subject [33]. However, an assessment of the knowledge of plagiarism did not find a significant difference between the students who were only doing medicine with those who had also been enrolled in MPH courses [65]. It is likely that the course does not sufficiently address ‘publication ethics’. A survey of 198 students found that medical interns (medical students during the last two years of their studies) seemed to know more about plagiarism than the sub-specialty residents [67]. This could show that either the residents belonged to the generation when not much ethics training was available, or the residents may had been trained before but there existed a need for repetition of the courses.

The reviewed literature proposed several reasons for the emergence of Iranian publications with traces of research and/or publication misconduct that are summarized as follows: (i) inadequate knowledge of plagiarism and of the regulations in place to deal with cases of plagiarism [43,56,64,65,67,68]; (ii) poor English language skills and limited writing skills that could lead to plagiarism [43,59,65,68]; (iii) requiring faculty members to have publications for academic promotion [43,56,69]; (iv) requiring PhD students to have publications before graduation [43,69]; (v) requesting certain academic degrees from people who apply for political positions [70,71]; (vi) installing powerful people as the heads of research centers may have led to guest authorships [72]; (vii) limited budget that cannot cover all research costs [69]; (viii) as a results of the rapid development of postgraduate programs, some individuals have quickly become faculty members, without having been appropriately trained to mentor students in research [43]; (ix) replacement of competent academics in Iranian universities by less qualified faculty members who were unaware of research activities and ethics regulations [70]; and (x) limited interaction between the academics and other sectors, leading to insufficient awareness of academics about the final goal of research, ie, filling the knowledge gaps to address the problems, rather than solely leading to publications [43].

#### Monitor and evaluate HRS

In 2000, the Department of Research and Technology of MOHME initiated an annual evaluation of research activities of all the universities and research centers affiliated with MOHME [31]; including 58 universities of medical sciences and 736 research centers in 2017 [73]. The results of the evaluation of each institution used to be reported to them as written feedback, while since four years ago, the results have been sharing online [26]. The strengths and the weaknesses of the academic performance at the levels of national and institutional are summarized through these evaluations which become the evidence to inform decision making at MOHME and at the institutions towards improving health-related research activities [74].

In this evaluation system that was initially designed based on the WHO HRS framework, research activities are assessed and scored against indicators across three domains: (i) capacity building; (ii) knowledge production; and (iii) stewardship (in recent years, this domain has been renamed to ‘leadership’) [30,31,75]. Although the indicators are revised every year based upon the feedback received from research directors of the universities and research centers [74], they generally continue to include the points described below.

For the assessment of capacity building in each institution, the indicators include the number of: (i) research training courses provided to the academic staff; (ii) national and international conferences organized by each institution; (iii) awards that had been achieved by the staff/students at science festivals; (iv) status of the amount and the visibility of the institution’s Web contents; and (v) the state of Student Research Committees (SRCs) [76]. The second domain, ie, Knowledge production, is evaluated by the number of: (i) journal publications (the scores allocated to the publications vary according to the databases where the publications are abstracted, eg, in Scopus, Web of Science Core Collection); (ii) abstracts presented at national and/or international conferences; (iii) published books; (iv) patents; (v) completed research projects that their results had been applied in the health system; and (vi) citations to the published papers in textbooks and peer-reviewed journals [31]. Stewardship domain includes the following: (i) having had identified institutional-level research priorities and the status of adherence to them; (ii) having a 5-year strategic plan; and (iii) having an active ethics committee [30].

The universities have been categorized into three groups, and they are ranked within each group based on the total score they receive from the three domains mentioned above [31]. The criteria for grouping the universities were not clearly defined in the literature, although it seems to be according to the universities’ general size, without further definition [75]. In the first years since the evaluation was introduced, the scores used to be adjusted by the amount of core funding and the number of academic staff at each university [31,75]. In recent reports, the scores do not seem to be adjusted anymore [31,75]. Furthermore, according to the literature, institutions used to receive scores for collaborative research with industry and/or the governmental organizations and for multi-center projects [21]; these seem to have been removed in the recent evaluations, although international collaboration still contributes with additional points [30,74]. Several citation-based indicators had been added to the assessment of “knowledge production” domain in recent years, eg, citation counts per paper, h-index of institutions, or impact factor (IF) of the journals where the articles had been published [75].

Although this system has allowed the annual evaluation of health-related research activities in Iran, it has been criticized for its over-reliance on quantitative indicators; lacking qualitative assessment by a panel of experts [77]; and minimal attention to research impact [78]. A pilot study in 2015 [78] attempted to include assessment of the impact and the quality of research activities in the evaluation of research centers. It evaluated the research activities of 5 biomedical and 3 clinical research centers using a peer review approach [78]. Indicators were designed for four domains: governance and leadership, structure, knowledge production, and research impact. The implementers of that pilot study concluded that the peer review model would work for the evaluation of research output, outcome, and impact of medical research centers in Iran [78].

#### Stewardship – Discussing the main points

Iran has successfully developed necessary structures for both formulating and implementing health research policies. However, perhaps too often the policies are not well implemented because of several constraints, such as the absence of qualified people at executive roles. Stewardship seems to be the Achilles heel of the HRS of other countries in the WHO Eastern Mediterranean Region (EMR) too [6]. In 2008, it was reported that [79] in a sample of 10 EMR countries, only four had structures for national governance of HRS, and just two countries had national health research policies [79].

This review also found multiple exercises that had attempted to identify the health research priorities although a need for more systematic, inclusive, and less time-consuming approaches to research prioritization was evident. Global literature shows that lacking systematic and transparent priority-setting exercises is a mutual problem of the HRSs in most LMICs [80,81]. Moreover, it has been reported that the majority of health research priority-setting exercises in LMICs have failed to engage the key stakeholders (eg, the community) in the processes, and they have heavily relied on the input from researchers and representatives from the governments [80,82].

The reviewed evidence show that research and publication integrity in Iran’s academia still require significant improvements. Of the identified obstacles for improvement, overcoming some of them requires fundamental changes. Examples of this group are to improve meritocracy in recruiting faculty members as they train the future researchers; or changing the education system, perhaps from primary school, to improve the level of English and writing skills of the graduates. On the other hand, addressing some other constraints seem more feasible in medium term, such as modifying the regulations that mandate academics and students to publish papers; provision of further training on how to avoid and address plagiarism; and improving interaction between academia and the end-users of research.

Finally, it is promising that a system is in place for monitoring health research activities of academic institutions in Iran although for ranking purposes there seems to be a demand for: adjustment of the scores by the input of each unit (eg, the core funding); less reliance on bibliometric indicators; further attention towards the quality and the impact of research; and additional rewards for collaborative projects between academia and the users of research.

### Financing

The reviewed literature generally suggests that investing in health research has not been a priority in Iran. This is reflected in Iran’s Gross Domestic Expenditure on Research and Development (GERD). Iran’s GERD for nine specific calendar years were found in the reviewed literature: it ranged between 0.31% (in 2011 and 2014) [15] and 0.75% (in 2007) [6,77,83]. Although GERD has remained limited, the annual budget that MOHME had been allocating per research center had increased more than 80 times between 2001 and 2014 (from 4.8 million to 387.5 million Iranian rials per center) [84]. It was also reported that the amount of research budget per academic member in institutions affiliated to the government had increased by five times between 2002 and 2010 [30]. However, these two reports of the increase should be considered cautiously as the amount of investments was not adjusted by the annual inflation rate.

It is also important to note what proportion of GERD is allocated to health research. This figure had decreased from 8.9% in 1999 to 7.6% in 2001 [21]. Another publication in 2001 had reported that 5% of GERD was invested in health research [83]. Regarding the proportion of GDP that is allocated to health research, the figures ranged between 0.01% to 0.05% between 1991 and 2001 [18], while funding for health research as a proportion of the total health care budget was 0.9% and 2.5% in 1991 and 2001 [21]. No information from the recent years was found in the reviewed documents. It was suggested that the integration of medical education and health research with health care services, which took place in 1985, may have shifted resources away from research to service provision [85]. **Box 4** provides further information about the integration.

Box 4 **Integration of medical education and research into health services**

In the 1980s, Iran suffered from a severe shortage of health care professionals. In some parts of the country, there was only one physician to provide services to a population of over 18 000 [86]. To tackle this problem, in 1985, “medical education and research” were integrated into “health services”, aiming to increase admissions to medical universities [87]. This integration led to the creation of the Ministry of Health and Medical Education (MOHME) [85]. The main aim of the integration was achieved a decade later: in 1994, the number of medical students had increased to 5.8 times in comparison to 1970 [85]. This organizational reform eventually resulted in the universities in the provinces taking over all the activities related to medical education, research, and provision of health services in the capital of each province, its urban, and rural areas [85]. The chancellor of the university in each province became responsible for these activities [85]. In the short term, this led to the training of a substantial number of health care professionals and the development of health services across various parts of the country, particularly in deprived areas [88]. In the longer term, the integration has increased the universities’ workload and responsibilities [85]. It is reported that universities now invest more time and resources into providing service than on research and education [85].

Some minor progress has been made in financial aspects of health research in Iran. For instance, since 1996, GERD began to be specified in Iran’s annual national budget plan, which has relatively improved transparency in financing and has allowed monitoring the investments in research [83]. Furthermore, several papers have reported that, in recent years, some major macro-policy documents had set targets for substantial rises in R&D expenditure, although the targets were not eventually met [15,89]. An apparent example of failure was the target of increasing GERD to 2.5% by 2015, whereas the last retrieved GERD was only 0.3% (in 2014) [15]. It was suggested that the failure in increasing expenditure in R&D is in policy-makers' lack of belief in the return of investment in research [89]. Finally, it was mentioned that insufficient financial resources for health research have led, at times, to the complete exclusion or underfunding of specific areas in national-level studies, such as mental health research [90].

Transparent information on the mechanisms of distribution of health research funds was absent in the retrieved documents. It was mentioned that, in 2011 [15], more than 80% of GERD was distributed through the universities and institutions affiliated to MOHME but also to the Ministry of Science, Research and Technology; Ministry of Defense; Ministry of Industry; Ministry of Agriculture; Science and Technology Parks; and institutions affiliated to the Vice-Presidency for Science and Technology. The latter is a group of research councils under the supervision of the Presidential Office [15]. First, no information to estimate the share of each of these entities of the total funds was provided. Second, the sustainability of funding decisions within this model of distribution – which is predominantly through the governmental organizations – was criticized for being vulnerable to the opinion of politicians who are changed every few years in Iran [91].

In yet another example of insufficient information on how the funds are distributed, in 2015, there were 36 medical research centers earmarked in the national budget plan for receiving funds directly from the public budget [84], but no information regarding the criteria upon which the research centers would be earmarked was provided. Also, it was implied from the reviewed literature that health research funds are somehow distributed equally among academics who work in the institutions affiliated to MOHME [30]. Finally, it was reported that in 2013, 40% of health research budget was invested in ‘research projects’, 70% of which were in line with the institutional-level research priorities [30]. It was unclear what the authors meant by investment in ‘research projects’ and it was not mentioned where the remaining 60% of the health research budget was invested. For the period 1997-2001, it was reported that 5.76% of the total public funding for health research had been allocated to research priorities in the health system [21]. In sum, a system for tracking research investments seemed to exist, although its function was not described.

In terms of the source of the research budget, the public sector has remained the primary source of research funds in all fields in Iran, including health [15,21,92]. In a 2004 report by the WHO, the share of non-governmental sources (ie, private companies, scientific associations, and NGOs) was estimated to amount to 3%-6% of the total funds for health research in Iran, and the figure had decreased from 5.9% in 1991 to 2.7% in 2001 [21]. Some have reported that, in recent years, the contribution of the private sector to research investment has increased. For instance, in 1998, less than 1% of investments in R&D was from non-governmental sources [15,83], whereas in 2009, nearly 31% was the contribution of the private sector [15]. Nevertheless, it was reported in 2016 that public funds still constituted 98.5% of the research budget of medical research centers in Iran [84]. A paper investigated the status of collaborations in research projects that were conducted between 2005 and 2007 in one of Iran’s universities (Golestan University of Medical Sciences) and found that among the 102 included studies, only 12 of them (11.8%) had been co-funded by organizations outside the university [28].

#### Financing – Discussing the main points

One basic step to improve financing of the HRS, which Iran has already taken, should be to ensure that the official budget plans allocate a proportion to R&D – no matter how small. Still, the literature suggests that investing in health research has never become a priority in Iran. Iran’s R&D expenditure has even been decreasing in recent years (reaching only 0.3% in 2014). This downward trend is in contrary to the ambitious figures that are envisioned for GERD in some of Iran’s major macro-policy documents. Allocating limited financial resources to research is a common problem across the majority of LMICs [2] and most parts of the EMR [6]. Exceptions in the EMR are a few Gulf countries that have been increasing their research investments in recent years [6]. For instance, GERD in Saudi Arabia suddenly increased from only 0.04% in 2008 to 0.8% in 2010 [93]. However, these countries have a quite weak national capacity for health research and, hence, their research highly depends on international academics [6]. For example, according to the data abstracted in Scopus database, 75.8% of Saudi Arabia’s publications in 2017 had international collaborators [94].

While transparent information on the mechanisms of distribution of research funds was absent in the retrieved documents, it was implied that health research funds are somehow distributed equally among academics. Such a mechanism does not seem appropriate in a country where research budget is limited and mainly dependent on the government, while receiving funds from international sources is rare too. However, distributing equal funds among academics seems aligned with the egalitarian values of the 1979 Islamic Revolution which perhaps had aimed to make education and research universal all across the country [10]. While this approach could provide equal opportunities for all to partake in research activities, distribution of equal funds within this system could leave insufficient resources for the academics with potentials to conduct high-quality research which often demand larger funds. Furthermore, in medical fields, it seems that the individual-level financial incentives for clinical and or teaching activities are far more than that for health research activities in Iranian universities of medical sciences. An investigation of the institutional constraints on the Iranian academics in non-medical science fields had reported that the outstanding academics were the most frustrated ones, while the mediocre researchers seemed rather satisfied with their career in Iran [95].

### Creating and sustaining resources

Over the last five decades, Iran has largely developed its higher education capacity in medical sciences. This development is reflected in the substantial increase in the figures for physical (infrastructural) resources (eg, the number of schools of medicine) and human resources. As will be reviewed in this section, the efforts towards expanding the capacity of medical education primarily aimed to increase the number of health care professionals all across the country to address the shortage of physicians. However, this already existing human capital was later enabled and encouraged to engage with research activities, too.

#### Changes in the figures for human and infrastructural resources

**Figure 1:**
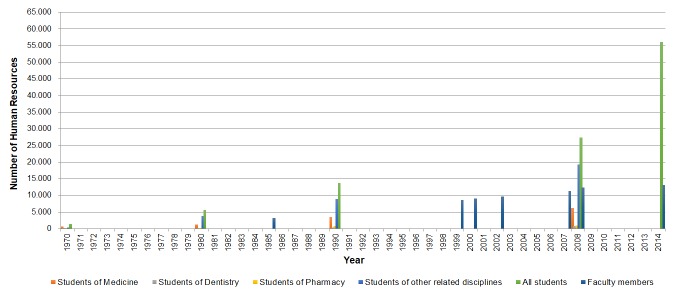
The number of Iran's human resources in academic institutions in medical sciences, 1970-2014.

While there was inconsistency in the figures across the reviewed documents, all the retrieved data reported a significant rise in the number of academic staff and students at the universities of medical sciences affiliated to MOHME, at different levels of education and training (ie, primary qualifications, specialty, sub-specialty, and postgraduate programs) [15,83,86]. **Figure 1** highlights that the literature lacked data on human resources in most years while indicates the significant rise that has occurred in the number of both the students (during 1970-2008) and academic staff (during 1985-2014). The substantial increase in the number of students in disciplines related to medical and health sciences started in the late 1980s [86] and had continued, reaching 56 131 in 2014 [15,30]. It was also mentioned that over the last few decades, the proportion of female students in higher education has greatly increased; for instance, the percentage of female students increased from 42% in 1990 to 68% in 2013 [15].

Investigating the number of faculty members in different years was rather confusing. Certain terms, eg, “teaching staff”, “academic researchers”, or “non-academic researchers”, were used without being defined [15]. Moreover, it was often unclear whether the reported figures for faculty members included or excluded the academic staff of medical universities that are not affiliated with MOHME, eg, the academics employed by private universities or medical schools affiliated to military organizations. The information summarized here (shown in **Figure 1**) are from the sources that had appropriately described the figures. The number of faculty members in the universities and/or research centers affiliated with MOHME had increased from 3153 in 1985 [49] to 8625 in 1999 [83], 11 324 in 2007 [31], and over 13 200 by 2014 [15]. It was mentioned that in 1999, there were also 1158 faculty members in the universities outside the structure of MOHME [83]. It was also noted that a new type of academic position was introduced in 2010 as “research-focused faculty member”, meaning some academic staff were recruited for positions which did not involve any teaching; in 2010, 289 of them were recruited [30].

The increases in the number of specialty, sub-specialty, and PhD programs were also highlighted in the literature. Until 1980, Iranian medical universities offered only a few specialty programs, no sub-specialty, nor PhD programs [86]. It had been reported that between 1975 and 2008, the annual number of admissions to specialty programs increased from 401 to 1732 students, and from zero to 268 in sub-specialty programs [86]. A significant increase in the number of students enrolled in masters and PhD programs in all disciplines was reported between 1998 and 2013: the first one had increased from 23 303 to 454 978 and the latter from 3771 to 60 900 (more than 16-fold increase) [15]. While the figures for the programs relevant to health sciences were not specified in that paper, another report showed the rise in the total number of students admitted in doctorate and masters programs with a health research component for the period 1997-2001 from 44 to 216 [21].

In 2010, MOHME launched a new scheme of PhD programs, so-called “PhD-by-research” [15]. In 2017, 616 students were enrolled in these PhD programs across 188 research centers affiliated to MOHME [15]. Unlike the conventional type of PhD programs in Iran, where students are awarded positions only after excellent performance at an annual national entry exam that relies on multiple-choice questions, “PhD-by-research” students are assessed, recruited, and supervised by faculty members whose eligibility is approved by MOHME [96]. Also, while one of the criteria for being offered a “PhD-by-research” position is having a certain number of publications in international bibliographic databases, graduation from these programs is also by publication [96].

It was reported that because of the significant increase in university admissions in the 1980s, initially, the ratio of students to faculty members had increased [86]. Later on, consequent to the development of postgraduate programs, the number of individuals who were qualified as faculty members also increased, so that the ratio relatively improved [49,86]: from 17.1 students per faculty member (in 1985) to 8 in more recent years [83,86]. Finally, the number of employed researchers in research centers affiliated to MOHME has risen from 637 in 2001, then 3828 in 2010, to reach 5736 in 2014. This translated to an average of 10 researchers per research center [84] (although the definition of “researcher” was not fully explained).

Regarding infrastructural resources, the number of academic medical institutions affiliated to MOHME has substantially increased [73,83,84]. This includes the increase in the number of universities of medical sciences from 34 in 1996 to 58 in 2017 [73]. Also, between 1970 and 2008, the number of schools of medicine had increased from 7 to 36; and schools of dentistry and pharmacy from 3 each to 15 and 11, respectively [86]. Likewise, the number of schools of nutrition, public health, nursing, midwifery, and several paramedical disciplines has significantly increased [86]. Over the last two decades, a substantial increase has taken place in the number of research centers in areas related to medical sciences: from only one center in 1992 to 53 in 2001, 359 in 2010, and 736 in 2016 [73,83,84]. The majority of medical research centers are reported to be in the areas relevant to internal medicine, pharmaceutical sciences, and cellular biology [86]. Research center was defined as “a facility or building dedicated to research, commonly with the focus on a specific area” with no further requirements [73]. It was reported that the increase in the number of research centers between 2010 and 2016 occurred without sufficient oversight and led to some challenges [73].

#### Capacity building

Short-term and long-term strategies have been adopted to build capacity in Iranian academics in areas related to health research. First, many students have obtained research-relevant training during postgraduate studies. Then, numerous training courses relevant to research have been offered since 1990 by the MOHME’s affiliated universities and research centers [18]. The number and diversity of these courses – from basic research methods to statistics, academic writing, and research methods that are used in specific fields, eg, in mental health – have been increasing over the last decades [90,97,98]. The figures had increased from 458 in 2000 to 1097 in 2007 [31]. A survey in 2004 showed that the majority of participants were satisfied with the quality of the courses that had been provided since the early 2000s [21]. More recently, research centers have been increasingly providing similar courses; the number of workshops organized by medical research centers increased from 92 in 2001 to 625 in 2014 [84]. Furthermore, some research hubs that have achieved a reasonable capacity for research in specific areas are now building capacity in their peer institutions. For instance, Royan, a leading Iranian stem cell research center, now provides courses related to research in stem cell and tissue engineering [39].

The role of Student Research Committees (SRCs) was also noted in the literature in developing research capacity. SRCs are run by students and they promote research among the students of medical sciences primarily by: (i) offering research methods training; (ii) conducting research; (iii) writing papers; and (iv) presenting at conferences [31]. Some SRCs even raise funds to sponsor travels of the committee members to international conferences [99]. Finally, SRC members try to acquire other essential skills for research, eg, communication, management, and teamwork [100]. The first SRC was established in TUMS in 1993 by a group of enthusiastic undergraduate students who aimed to create a supportive and enabling research environment at universities [100]. Later on, under the supervision of the Department of Research and Technology of MOHME, SRCs were formed in all universities [31,99].

It was reported that a significant capacity has been built in certain disciplines, such as gastroenterology and hepatology, which have advanced substantially in educating and training clinical and research fellows [86]. It was suggested that such success could be related to the hard work and the determination of some devoted academics in those fields, who were also supported and provided with resources both by the governmental and non-governmental entities [86].

#### Barriers to research activities

Despite efforts to improve the capacity of Iranian academics in research and publishing, the number of skilled researchers in the country is still limited [18].An assessment of the knowledge, attitude, and practice of 436 students in an Iranian university of medical sciences in 2013 found that students' skills in using research methods were considered moderate; the majority had no positive attitude towards research activities; and their research performance was graded as weak [101]. Several papers have touched on the possible reasons. One hypothesis is that many motivated young graduates who have acquired research skills by attending courses and/or through working in SRCs during undergraduate studies would immigrate to developed countries soon after publishing a few papers and improving their academic CV [6,102]. Another proposed reason is that the students who join SRCs, or the faculty members who attend research-related courses, are likely to be already willing to improve their research skills [65]. Hence, research capacity in those with less interest in research may never improve much because research methods are not included in the core curriculum of programs in medical sciences and are only offered as optional courses[100].

Furthermore, the literature highlighted that the education curriculum in Iran does not equip graduates with the necessary skills for research. The medical education curriculum was particularly criticized for being too oriented towards the students passively learning facts [103], rather than looking for critical thinking or creative problem-solving. The model of admittance to Iranian universities was criticized, too, for being largely dependent on the performance of applicants on a competitive multiple-choice annual exam, which trains students to memorize facts instead of being critical thinkers [23].It was also emphasized that students in Iran’s higher education programs do not receive training to develop skills in communication, writing, management, or teamwork, which are requisite for becoming a competent researcher [23,103].

Another critical barrier to more enthusiasm for research are lower financial incentives for research compared to teaching or clinical activities. In an assessment of 186 academics from one Iranian university (Guilan University of Medical Sciences), 70% reported limited financial reward as a major constraint on doing research [23]. On the other hand, the extra payment that academics receive for additional teaching hours is often higher than earnings from their time invested in research [23]. Importantly, since in Iran, medical education and research are merged with health care services provision, many of the academics in the universities affiliated with MOHME are clinicians too. These clinical academics are paid greater salaries by working in teaching hospitals [23]. They even receive extra payment per patient that they visit in university clinics [23]. Therefore, they can have a much higher income through providing clinical services instead of investing in research activities [23]. A Commentary had suggested that many Iranian clinical academics still think of research as *a luxury good* while considering clinical activities as *a necessity good*. Thus, they engage with research only to get academic promotion, rather than contributing to the society or to the industry [104]. Also, one of the reported constraints cited the researchers’ own low expectation that their findings would be applied in practice [21].

Other constraints reported in a number of studies are summarized as follows: inability or unwillingness for collaborative work; lack of essential means and facilities to conduct research; restrictive administrative regulations; lack of university autonomy; limited organizational support and poor cooperation between executive offices within the universities; weak project management skills; inadequate number of qualified senior researchers to provide effective supervision; and limited number of qualified librarians [21,105-107]. On the individual level, heavy workload and limited time for research; poor knowledge of research methods and statistics; insufficient incentives; and inadequate support for academics with family commitments were mentioned as some of the barriers to medical and health sciences researchers in Iran [105,107].

Some literature argue that the international trade sanctions against Iran, which aimed to restrict Iran's nuclear program by targeting its oil and gas export, banking, and financial sectors, had posed another constraint on research activities of Iranians [102,108-110]. The reasons behind this argument are that the sanctions had (i) restricted exchange of Iranian students and faculty members with international academic institutions; (ii) made purchase of laboratory equipment and material difficult; (iii) negatively affected international collaboration; and (iv) at times led to the outright rejection of Iranian research papers by some journals [102,108-110]. In terms of the last one, in April 2013, Elsevier advised its US editors against handling any papers authored by employees of the Iranian government, which could include any academic working at the universities affiliated with MOHME, and this “advice” was followed by some journal editors [109,110].

On the other hand, although sanctions must have had made it harder for Iranians to conduct research and to partake in the international scientific community, data retrieved from international bibliographic databases show that the quantitative growth of Iran’s research publications had continued during the sanctions [102]. Also, as for publishing research papers with international collaborators, between 2005 and 2014 (ie, during tight trade sanctions), the rate of the growth of international collaboration in Iran was similar to that in Egypt and Israel, and higher than that in Turkey, suggesting that sanctions against Iran did not much affect its international scientific collaborations [111]. Likewise, the number of collaborations with the United Nations’ (UN) agencies showed no significant change during this period [84]. It was explained that perhaps the development of Iranian journals and the support of some of the main actors of health research in the international community, eg, the editors of *The Lancet*, might have alleviated some of the consequences of sanctions on Iranian research publications [111].

Finally, it was argued that one barrier against innovative and high-quality original research in Iran is that higher education opportunities are still not equally provided to all Iranians [112]. As an example of inequality, it was claimed that in Iran women are banned from studying 77 disciplines (without providing the list) and that equal opportunities may not be provided to all religious minorities [112].

#### Collaboration

Collaborations and coordinated activities are crucial to sustaining and strengthening research resources [6]. In the early 2000s, the Department of Research and Technology of MOHME introduced several initiatives to promote collaboration in research. One initiative was that, between 2001 and 2011, MOHME signed official memorandums for collaboration between Iran and academic institutions in Sweden, Germany, South Africa, Belarus, Malaysia, Indonesia, and the Eastern Mediterranean Regional Office of the WHO [113]. These memorandums led to the joint training of 20 students; 18 collaborative projects; 26 publications; and co-organizing 17 workshops [113]. The second initiative, beginning in 2001, was for expansion of collaboration between researchers and the community [21,114]. This initiative was implemented through the establishment of community-based participatory research centers in several medical universities across Iran. These centers supported the conducting of projects that could lead to capacity-building in the community so that the community’s knowledge could be used for addressing health problems, particularly those regarding the social determinants of health [114].

The third initiative of MOHME towards promoting collaboration was the development of knowledge networks, aiming to organize, lead, empower and coordinate efforts made by health researchers and key stakeholders to: first, prevent repetitive and/or parallel health research in Iran; and second, strengthen knowledge translation [21,115,116]. By 2012, Iran had 27 knowledge networks in medical and health sciences [116]. However, it was shown that the approaches used in the management of those networks was not very transparent and that the majority lacked clearly defined goals and faced multiple administrative problems [115]. Still, according to the literature, some networks – eg, Iranian Osteoporosis Research Network (IORN) and Iranian National Diabetes Research Network (INDIRAN), both established in 2002 – made significant contributions. IORN, by 2008, had linked 21 Iranian universities and research centers and had: established osteoporosis clinics; initiated a multi-center osteoporosis study and a hip fracture registry project; and run education and prevention programs [117]. By that time, INDIRAN had also completed and/or initiated multiple projects, eg, estimating the prevalence and the burden of diabetes in Iran; evaluation of the quality of life in diabetic patients; and running diabetes education and prevention programs [118]. It was mentioned that, despite accomplishments of INDIRAN, some provinces still required research facilities, trained researchers or research centers in diabetes to be able to collaborate on national projects [118].

To further promote collaborative research, the Department of Research and Technology of MOHME that runs a national, annual academic assessment, by which it ranks the Iranian medical universities and their affiliated research centers, allocated points to collaborative research projects that engaged several institutions [23]. However, the regulations that were in place for assessment of academics still provided more points to single-authored papers, which discourages collaboration. The regulations and requirements for academic promotion are set in a different organization. This conflict in regulations suggests that further coordination is required for the success of initiatives that promote collaboration [23].

Despite the abovementioned efforts, the literature suggests that the status of research collaboration is not satisfactory. A paper investigated the state of collaborations in research projects that were approved between 2005 and 2007 by an Iranian medical university (Golestan University of Medical Sciences) [28]. Among the 102 assessed studies, only 10 projects (9.8%) had been undertaken in collaboration with other organizations; and only one project was commissioned by an NGO [28]. Another study showed that of the 208 research projects conducted in TUMS in 2004, only 2.2% had a partner from non-academic organizations, and 51 researchers (24.5%) stated the users had not contributed to any stage of the research [85]. A qualitative study in 2004 investigated the status of collaboration between universities of medical sciences and their affiliated research centers with either the community or the executive organizations and found the following: 20% of the academic institutions had no link with the private sector, while collaboration with the community as well as with executive entities was weak too, particularly in knowledge utilization and identification of research priorities [119].

#### Creating and sustaining resources – Discussing the main points

In sum, over the last five decades, Iran has largely developed its higher education capacity in medical sciences, as reflected in the increased number of universities of medical sciences and schools and research centers within the universities, as well as the substantial rise in the figures for academic staff and students. It was reported that some of these increases have occurred without sufficient oversight or without having had the essential capacity (eg, inadequate number of qualified mentors to supervise the large number of recruited PhD students).

In terms of capacity-building, numerous training courses relevant to health research have been offered since 1990. However, the number of skilled researchers in the country is still limited. It was emphasized that students in Iran’s higher education programs do not receive training to develop skills in communication, writing, management, or teamwork, which are necessary for becoming competent researchers. More importantly, it seems that the majority of the research trainings have focused on research methods, or on teaching some “techniques” to write and publish research papers. There is a lack of an environment which educates students and academics about *why to do research* and *why to publish it*.

Some have corresponded the small number of skilled researchers in Iran to Iran’s high rate of brain drain: approximately 150 000 Iranian specialists emigrate every year [120]. Many LMICs, despite having research-intensive universities, lose their trained human resources to the brain drain [6,17].This is because in addition to availability of good academic institutions and facilities to undertake health research, a favorable research environment, encouraging remuneration and career prospects, sufficient research funds, and opportunities to openly discuss research should exist to encourage talented individuals to stay [6,17].

To encourage the return of the Iranian diaspora, particularly in science, the current President (2013 – present) has made several efforts [120]. Although figures on the outcome of the government’s efforts are unavailable, sporadic reports show no significant success in this regard [121]. It should be highlighted that inviting back the diaspora is only one way to address brain drain. Finding other ways to connect with diaspora and their international networks is critical. Many emigrated scientists are often willing to “informally” collaborate with their peers back home, by providing intellectual, technical, or material assistance [8,17]. The author has provided some examples of successful collaborations of overseas Iranians with academics based in Iranian institutions elsewhere [14]. Given the international isolation of Iran which has declined “formal” exchange of academics between Iran and international institutions and has restricted Iranians’ access to international research grants, establishing and maintaining “informal” collaborations with the Iranians abroad seems more feasible.

### Producing, disseminating, and using research

#### Producing and disseminating research

A substantial rise over the last few decades has been reported in the number of Iranian health-related research papers in international bibliographic databases [15,76]. For instance, it was reported that the number of Iranian documents in MEDLINE had increased from only 273 in 2000 to 14 511 in 2014 [15]. This review retrieved 34 papers that had reported the quantitative growth of Iranian publications in different biomedical, clinical, and/or public health research areas (Summarized in Table S2 in **Online Supplementary Document(Online Supplementary Document)**). It was also noted that the rise in research output had occurred regardless of the population growth. For instance, the number of publications per million Iranian inhabitants had increased from 155 in 2008 to 326 in 2014 [12,15], while the number of publications per academic has risen too [31]. The growth had also been observed in the figures for the Iranian papers in Persian (a 30-fold rise between 1979 and 2003) [86], and the number of Iranian books that have used the findings of national research had increased as well [76].

In terms of citation-based indicators, the number of citations that Iranian research papers have been receiving in medical textbooks [76] and the citation counts per academic have increased [15]. Also, Iranian papers are being published in journals with higher IFs than before [21,86]. Furthermore, in 2004, Iran was reported as one of the countries (and the only EMR country) that had contributed to 98% of the world’s most-cited publications [6].

Likewise, the number of Iranian medical journals, both national and international, has increased over the last few decades [31]. Only between 1990 and 2010, 155 new medical journals were approved by Iran’s MOHME and many of them have found their way into international bibliographic databases [91]. Improvements have also been reported in the citation-based indicators of the Iranian medical journals that are indexed in the Web of Science Core Collection: eg, between 2012 and 2014, their average IF had increased from 0.40 to 0.68 and the average number of citations that each of the documents in these journals has been receiving from international authors had risen from 0.19 to 0.49 [122]. However, Iranian journals have been criticized for being predominantly published by the universities; it was argued that this closeness of journals to where the research originates could have been negatively affecting the independence of peer review processes [91,123].

While the increasing number of Iranian journals that are indexed in international databases has significantly improved the visibility of Iranian publications [11], the publications in the national journals do not yet seem to be very applicable by the international readers [124]. An assessment of the web-based databases where Iranian journals were indexed has found that none had a complete coverage of Iranian journals; the search features were sub-optimal; English translation of the titles of the Persian papers, authors’ names, keywords, and abstracts were not aligned with standard formatting styles; there were numerous typos in the English content; and some websites did not even provide English abstracts [124]. This assessment had included the following websites: IranDoc; IranMedex; MagIran; Scientific Information Database (SID); and Shiraz Regional Library of Sciences and Technology [124].

In terms of the substantial growth of Iranian health-related research publications, although no explanatory study had investigated the national-level contributors to the growth, several possible reasons were hypothesized in the reviewed papers as follows: (i) increased investment of the government in R&D [11,12,74] and increased investment in the health sector[23]; (ii) increased number of research centers and medical universities [12,23,71]; (iii) increased number of faculty members [11,23]; (iv) providing academics and students with training courses in research and publishing [74]; (v) introduction of regulations that required faculty members to have papers for academic promotion [59,91,104]; (vi) increased number of students [11]; (vii) the large number of young and talented researchers who mostly publish papers to improve their academic CVs [102]; (viii) introduction of regulations that required PhD students to have papers abstracted in international bibliographic databases [11,43]; (ix) improved quality of Iranian journals and increased number of Iranian journals that are indexed in international citation databases, eg, Scopus [11]; and (x) increased international collaboration, which may have been for bypassing the international sanctions [71,111].

Regarding the institutional-level underlying reasons for the growth, one paper in 2014 studied the success of implemented policies in one major Iranian university (Shahid Beheshti University of Medical Sciences) towards promoting research and publication among its academics [125]. They found the following interventions that seemed to have had contributed to improving the quantity, the quality, and utilization of research [125]: (i) providing research courses for administrative staff so that they could speed up the research grants’ allocation processes; (ii) expanding subscription to international journals and bibliographic databases; (iii) equipping a laboratory with facilities to run PCR (ie, polymerase chain reaction) tests and do MRI on laboratory animals; (iv) establishing a center to serve as an intermediary between the departments of pharmacy and pharmaceutical manufacturers to help with knowledge translation and raising research funds from the industry; (v) replacing the paper-based administrative processes of research with online forms; (vi) identifying research priorities and encouraging adherence to them; (vii) increasing (more than doubling) the number of research centers of the university; (viii) introducing grant schemes specific to prolific authors (the amount of the awarded grants was dependent on the indexing and the IF of the journal where the previous papers of the academics had been published); and (ix) introducing a regulation that research publications were requisite for remuneration [125].

Regarding the quality of research, the literature suggests that still most of the output is not of high quality [18] although in most cases the quality was only assessed by citation-based indicators [69], a practice which is widely criticized [16,126,127]. A quality assessment of 509 Iranian clinical trials published during 2008-2010 in national journals reported that the adherence of 43.8% of the publications to the standard CONSORT checklist was inadequate [128]. Nonetheless, regarding reporting, an analysis of 795 clinical and/or health systems research articles published between 2001 and 2006 that had included Iranian populations on maternal care, diabetes and tuberculosis (indexed in national and international databases) found that 98.5% of the papers contained a clear message [129]. One study used two standard tools for quality evaluation of the methodology and reporting in Iranian papers in medical education research (2003-2008) that were abstracted in MEDLINE, and/or SID [130]. The study suggested that the quality of publications was suboptimal, particularly the problems were regarding the validity of research methods and the reporting of study limitations [130].

#### Using research

In terms of knowledge translation (KT), according to the literature, several steps have been taken in Iran over the last recent decades which seem to have been effective in the promotion of KT. The activities could be grouped into the categories of *Supply* of knowledge which is relevant to the users; *Demand* for knowledge; and *Exchange* – which includes the interaction between knowledge producers and users [6,92]. In the *Supply* category, while capacity has been built in academia to increase needs-based research, KT activities had also been incentivized [92]. Regarding capacity building, representatives from almost all Iranian medical universities participated in a KT training course in 2009 that was organized by the WHO EMR Office; the course covered topics from the basics of KT to the passive and active KT strategies [92]. In terms of incentives, the annual evaluation of academic institutions that is undertaken by MOHME allocates points to the implementation of research findings [31] and – likewise – according to the 2008 revision of the regulations for academic promotion, medical universities’ faculty members could receive points by KT activities [92].

In the *Demand* category, although some assume that the integration of medical education and research into the health care services provision has closed the gap between the knowledge producers and users in Iran’s health system [6,21], evidence suggests that this model has not much succeeded in this regard (Further details in **Box 4**) [85]. On the other hand, an attempt that seems to have been successful is that in recent years, the executive departments of medical universities were asked to allocate nearly 2% of their budget to ‘applied research’[21,92]. Hence, the university executive departments commission the academics and provide them with funds to address the needs of the executive departments through research [21,92]. Moreover, since 2005, MOHME has been calling for applied research grants, eg, in Health Systems Research. Finally, great efforts have been made to promote using the findings of systematic reviews and clinical trials and, in general, ‘evidence-based decision-making and practice’ among service providers and policy-makers [49,92].

Another attempt that improved *Demand* in KT was the development of the Iranian Registry of Clinical Trials (IRCT) in 2008 which increased the public accessibility to the ongoing and/or the findings of completed clinical trials in Iran [49]. The number of registered clinical trials in IRCT rapidly increased. It was more than quadrupled between 2009 and 2010 (rising from 181 to 772), presumably as a result of the regulations introduced by the Iranian medical journals that requested authors to include IRCT codes in their submitted manuscripts [131]. This regulation indeed led to a substantial number of trials getting registered retrospectively, particularly after completing the recruitment of participants; 62% of the registered trials in 2011 had been submitted after the end of patients’ recruitment [131].

In the *Exchange* category of KT activities, significant efforts have been made to identify health research priorities at the national and institutional levels with the engagement of the academics and the key stakeholders [92]. Further information on this subject is provided in section on identifying health research priorities and coordinating adherence to them and in Table S1 in **Online Supplementary Document(Online Supplementary Document)**. Also, as described in section on collaboration, community-based participatory research centers were established to facilitate interaction of the academics and the community in the process of research [92]. A major attempt in the *Exchange* category has been the establishment of “incubation centers” where researchers could present their ideas and/or findings that have a potential of commercialization to the businesses and could receive seed funding to further develop their work [15,92]. Moreover, establishing Science and Technology Parks and supporting young graduates to found knowledge enterprises have been among the strategies towards improving *Exchange* in KT [15,92].

Between 2010 and 2013, the number of incubation centers increased from 98 to 148, while the figures for knowledge enterprises had also risen from 2169 to 3400, and the number of Science and Technology Parks had reached 33, from 28 [15]. The figures for research staff of the parks had increased from 16 139 to 22 000 within the same period [15]. According to the UNESCO Science Report, the number of Iranian patents, in all areas, submitted to the United States Patent and Trademark Office, had increased from only 3 in 2008 to 43 in 2013 [132].

Few studies have attempted to assess the status of KT in health-related fields in Iran. Two assessments of medical universities and research centers have reported the lack of an appropriate KT environment in Iran’s academia; insufficiency of financial incentives; supportive regulations and facilities for KT activities; and limited opportunities for interaction between the academics and the knowledge users [133,134]. KT activities in diabetes were assessed in 2015 and the overall status had been described as “lower than ideal”, and several barriers at the macro and the meso levels were found against improvement of the status quo [135]. It was reported that the most commonly used KT activity by Iranian medical academics has been publishing research in journals, ie, a passive KT strategy [92], although engagement in active KT strategies was reported to be significantly higher in Health Systems Research projects [136].

A survey of medical interns at a teaching hospital affiliated with TUMS described the knowledge of the basic concepts of Evidence-Based Medicine in the majority of the respondents as insufficient although the interns were willing to receive training to learn about it [137]. Another survey enquired 319 general practitioners (GPs) in 2008 about whether they had updated their knowledge of diabetes over the preceding two years, and if so, which sources of information they had been using [138]. It was reported that a total of 38% of the GPs had not updated their knowledge, and the ones who had, mainly relied on Iranian journals in Persian, showing that clinical guidelines did not have any place as a source of information and/or practice [138]. The main barriers to the development and to the use of clinical practice guidelines in Iran were reported to be the lack of an evidence-based health care system and insufficient political support at the macro level [139].

A survey of 304 nurses working in teaching hospitals in 2003 reported the following: 80.6% had not been involved with the conduct of any research since qualifying as a nurse; 70% of those who had undertaken some research had done it as part of a course; about 30% had never used research in their practice; 44.3% read research once every 2 or 3 months or more frequently; and the main information sources that the majority relied on for practice were nursing textbooks and/or asking the nurse supervisors [140]. The barriers to research utilization in nursing practice in Iran had been identified as follows: (i) insufficient time to read papers; (ii) inadequate facilities and time to implement research; (iii) limited authority of nurses to change practice and limited cooperation of medical doctors in this regard; (iv) inaccessibility of scientist by whom the nurses could discuss relevant topics; and (v) inadequacy of relevant research [140-142].

The importance of “relevant research” was also highlighted in an editorial in 2007, where it was noted that, despite the substantial growth of mental health research publications in Iran between 1997 and 2002, the country still lacked the required evidence for national-level decision-making in mental health [143]. It was argued that this problem originated in the lack of a national policy that guides research investments towards mental health research priorities. Back then, conducting several systematic reviews was initiated to identify (i) the knowledge gaps, and (ii) the weaknesses in the quality of the earlier research that needed to be addressed [143]. Furthermore, an investigation of the views of 131 researchers and health research policy-makers on how the development and the usage of evidence from systematic reviews could be promoted in Iran had recommended: (i) the introduction of national-level initiatives for making systematic reviews ‘wanted’, and (ii) improving the capacity to conduct high-quality research [144].

Regarding the impact of medical and health research in Iran, most of the retrieved documents in this review – even the analyses that were commissioned by MOHME [78] – seemed to have been assessing the research impact solely by considering citation-based indicators. Only one study was found that had used a multi-dimensional approach; it had used the Payback Framework for impact assessment of a sample of 238 research projects that had been completed by 2008 [89]. The findings were as follows: half of the studies had published no articles in journals indexed in Scopus; the results of 12% of the studies had been used in systematic reviews; 12% had been used in clinical or public health guidelines; findings of only 5.3% had been used by MOHME in policy making; 62% were expected to directly lead to health impacts, of which only 38% had been implemented, and 60% had achieved the anticipated result; and of those with a potential of making an economic impact, nearly 36% had been implemented, of which 61% had made an economic impact, eg, by reducing the cost for the person and/or on the health system, and/or by reducing the number of work days missed due to illness/disability [89]. A rare example of research with an impact found in the literature was from Royan Institute, an Iranian stem cell research center approved by MOHME in 1998, where research is translated into medical services. It was reported that the center provides stem cell therapy for skin and cartilage disorders [145]. Finally, in 2003, it was reported that 13 national research studies had led to an improvement or change in the health system, while about 20 national guidelines for use in the health system had been reformulated based on the outcomes of locally conducted research (references to specific studies were not given)[21].

#### Creating and sustaining resources – Discussing the main points

On the one hand, several factors such as the increases in infrastructural and human resources, the introduction of policies that promote research, and the improved facilities for disseminating research have led to a substantial growth of health research publications in Iran. On the other hand, a vast amount of these publications do not seem very relevant to the needs of the end-users of research. The existing capacity could be better used with an improved research governance. There seems to be a need for fundamental changes to address the existing gap between the knowledge producers and users and to improve the use of research in decision makings and practice.

This paper had several limitations as explained in **Box 5**.

Box 5 **Limitations of this study**

This paper is subjected to the inherent limitations of a narrative review. One main limitation is that unlike systematic reviews for which there exist established guidelines to ensure rigor of the methods, there are no specific guidelines for conducting and reporting narrative reviews [20]. Nonetheless, there are some “best practice recommendations” to improve transparency and reproducibility of narrative reviews and reducing selection bias [20]. This is mostly done by employing an effective bibliographic search strategy and reporting it explicitly [20], which was performed in this paper. In terms of reporting, the structure of the Results and Discussion section was guided by the established HRS framework of the WHO, as described in the Methods.

Still, narrative reviews remain prone to bias as they represent the interpretation of the author from the existing literature. Some potential biases in this paper could result from the author’s past experiences as follows. The author before moving to the University of Edinburgh had obtained her first degree from TUMS (the leading medical university in Iran), had attended several research-relevant courses offered outside the main curriculum at her school, and had contributed to a number of clinical trials. Also at a smaller Iranian medical university, Mazandaran University of Medical Sciences, she had served both as a Research Associate and a medical journal editorial assistant (published in English). Furthermore, over the last three years, she has conducted several studies in relation to Iran’s HRS, two of which are published along this paper, under the same theme. Although the author has made substantial efforts to remain committed to the reviewed documents and evidence, her education and work background in addition to the knowledge generated in her other recent studies could have potentially led to some biases.

Finally, it should be noted that although this review conducted the search only in two databases, this should not be a dramatic limitation as one of the two databases was Google Scholar. Google Scholar indexes literally any scholarly literature that is available on the web [146]. Another concern could be that the search only used English keywords, which could have excluded the documents that were fully in Persian. One could argue that this might be the reason for the identified data gaps in many areas. However, it should be mentioned that several of the retrieved and reviewed documents were studies commissioned by MOHME for evaluating Iran’s HRS; this makes it quite unlikely that further relevant information could have been found in the public domain. A more comprehensive review may be possible through a documentary analysis which was outside the scope of this narrative review.

## CONCLUSION

This paper reviewed Iran’s evolution of HRS during the past 50 years. It bears essential lessons to share with three audiences: national-level health research decision-makers in Iran and other LMICs, as well as international stakeholders, eg, the WHO – to guide future health research capacity strengthening initiatives. The findings emphasized that improvement of HRS functions requires addressing context-specific problems. For instance, while formulating policies for the governance of health research is a critical component of the stewardship function of the HRS, having the policies in place will not suffice unless the barriers to the implementation of the policies are addressed. In this review, multiple examples were given of success stories regarding different functions of Iran’s HRS, that for instance, how one individual with links to influential decision-makers could promote medical research ethics in Iran. Or that how specific regulations in Iranian medical journals increased online accessibility to Iranian clinical trials. Several examples of context-specific challenges were found too, such as the barriers to research priority setting processes, and the obstacles that impede improving publication integrity. Reviewing the documents in the light of the world literature particularly triggered further thinking about possible approaches that can effectively strengthen HRS in contexts with a high dependence on very few sources of research funds, and centralized decision-making in HRS. Indeed, these problems are not exclusive to Iran, EMR, or LMICs, but could be found across the developed world too.

It was also concluded that Iran has built a great human resource capacity for research, a large number of research centers, and several medical journals that disseminate research originating in Iran and beyond. Nonetheless, the reviewed literature confirmed that research-intensive institutions should also offer an enabling and favorable environment and career prospects to encourage the trained academics to stay in the country. Some recommendations for improvement were increasing health research funds along with accountable allocation mechanisms; ensuring that the competent researchers receive enough resources to pursue high-quality research projects; and incentivizing research activities in medical universities as much as the clinical and teaching activities are supported. Finally, it was concluded that the identified barriers to knowledge utilization should be addressed so that high-quality and need-based research could be translated into policy and practice.
