# Supplementary material for: Evolution of Iran’s health research system over the past 50 years: a narrative review
Source: J Glob Health. 2018 Oct 25;8(2):020703. doi: 10.7189/jogh.08.020703 (PMC6204006; doi:10.7189/jogh.08.020703)
Supplement: Online Supplementary Document [file jogh-08-020703-s001.pdf]

Online Supplementary Document

Mansoori. Evolution of Iran's health research system over the past 50 years: a narrative review.

J Glob Health 2018;8:020703

Table S1. Health research priority-setting exercises in Iran over the last 10 years

Year	Brief description of the retrieved health research priority-setting studies
2008	Council on Health Research for Development's (COHRED) approach was used to set research priorities in infectious diseases [1]. All of the participants were from one university. The study identified 99 "research priorities" in 25 areas with HIV/AIDS, tuberculosis, and drugs being on top.
2009	To identify priorities in oncology nursing research, Delphi method was used during a regional conference although the study only involved Iranian nurses [2]. Among the 35 proposed areas, the top-3 priorities were as follows: (i) psycho-socio-economic impact of cancer diagnosis on family members; (ii) oral care in patients receiving chemotherapy; and (iii) nutritional needs of cancer patients.
2010	A modified version of COHRED was used to set research priorities at an institutional level by engaging 610 faculty members and 220 stakeholders [3]. A total of 841 research areas and 1,900 research options were identified as "priorities".
2010	An institutional-level study used COHRED to set priorities in a research centre of pediatric surgery [4]. It identified 7 areas of research and the highest priority was trauma in children, followed by pediatric cancers.
2011	Iran's research priorities to reduce burden of cancer were identified [5] by inviting cancer experts from all across Iran to rank a list of topics in cancer research based on the criteria of being necessary, appropriate, practical, and yielding in the Iranian societal context. An electronic system of communication was developed and all scientists were asked to rank each topic from 1 to 5. The results highlighted the need for prioritising studies on infrastructure of cancer control programs, cancer registration, service delivery, and patient quality of life.
2011	The research needs of a health insurance organisation was investigated through semi-structured interviews with 60 healthcare professionals who worked in clinical settings affiliated with the organisation [6]. Twelve research topics were proposed, among which 'Designing standard treatment protocols' scored highest.
2011	A Delphi study was undertaken for setting priorities in Health Systems Research at an institutional level for each of the departments of a medical university [7]. A total of 89 research areas were identified in their study which the top priority for each department varied.
2011	Research priorities in healthcare services were set using semi-structured interviews with patients who received services at three hospitals [8]. Thirteen research topics were identified of which studying the payment models that vary based on the patients' income and access to services was found as the top-priority.

2012	Research priorities in medical education for the countries in the Eastern Mediterranean Region (EMR), including Iran [9]. Using Delphi method a list of 20 research areas was proposed, of which the top area was 'training physicians to be effective teachers'.
2012	A national-level study used the Delphi method for identifying priorities in medical education research [10]. A total of 50 research topics were ranked in this national-level study of which the topic "methods for promoting faculty members' capabilities" was identified as the top priority for further study.
2013	Iran participated in a research prioritisation exercise, coordinated by the WHO Alliance for Health Policy and Systems Research [11]. The exercise identified research priorities for LMICs in health policy and systems research in the areas of access to medicines and used a mix of literature review, interviews with stakeholders, and reaching consensus. Eighteen research questions were formulated and ranked according to four criteria (innovation, impact on health and health systems, equity, and lack of research). The top research question was: "In risk protection schemes, which innovations and policies improve equitable access to and appropriate use of medicines, sustainability of the insurance system, and financial impact on the insured?".
2014	Research priorities in the field of patient safety in Iran were identified through a Delphi study where 45 research questions were rated and grouped [12].
2014	To identify the institutional-level health research priorities, the following methods were used: (i) semi-structured interviews with managers at a healthcare centre, (ii) questionnaires; and (iii) analytic hierarchy process for ranking the criteria [13]. This led to 191 research titles (as priorities) across seven themes.
2015	Using COHRED, institutional-level research priorities were identified for a research centre and a total of 31 research areas were identified as priorities [14].
2015	Health research priorities were set for one medical university by firstly, extracting a list of research areas from the goals and targets listed in macro policies, and secondly, inviting eight health research experts to rate the research areas based on COHRED criteria [15].
2016	Nominal Group Technique and Delphi were used to identify research priorities in the field of medical education at one medical university: Medical Ethics and professionalism gained the highest scores [16].
2016	A national-level study [17] invited experts to a workshop and asked them to list their suggestions for preventing invasive cervical cancer in Iran. After merging similar items and removing the duplicates, the experts were asked to rank the list of research suggestions. From the total of 26 suggestions, priorities were: developing national guidelines for cervical screening, and quality control protocol for patients' follow-up.
2016	Another study conducted a systematic review on published epidemiologic Iranian studies in HIV/AIDS and reported the knowledge gaps as research priorities [18].
2017	Delphi method was also used to determine health research priorities in occupational health in Iran [19]. It engaged 22 research centres across Iran and proposed that research in musculoskeletal disorders and injuries should be prioritized in Iran.

Table S2. A summary of the reported increase in the number of Iran's research publications in various research areas, using different data sources, and within different periods

Number of paper	Area	Data source(s)	Time period(s)
1	Medical education [20]	Index Medicus	1982-1998
2	Transplantation research [21]	data from 91 Iranian journals abstracted in IranMedex	1993-2003
3	Nephrology [22]	Medline and IranMedex	1997-2007
4	Nephrology and urology [23]	PubMed	1993-2013
5	Psychiatric disorders [24,25]	The national mental health data bank (IranPsych)	1973-2002
6	Substance use and addiction [26]	Web of Science, Medline, Scopus, SID, and Iranmedex	2008-2012
7	Epilepsy [27]	Scopus	2000-2014
8	Dental research [28]	Data from electronically accessible national journals and also through PubMed	1982-2006
9	Dental research [29]	Medline and IranMedex	1990-2009
10	Dentistry [30]	Web of Science	1993-2012
11	Dental research [31]	PubMed	2005-2014
12	Endodontic [32,33]	Pubmed	1992-2011
13	Orthodontic research [34]	PubMed, IranMedex, and SID	1997-2012
14	Mineral trioxide aggregate [35]	Pubmed	1993-2012
15	Trauma [36]	using the database of one of the largest trauma centres in Iran (Sina Trauma Data Bank)	2000-2006
16	Ophthalmology [37]	MEDLINE/PubMed	1981-2010
17	Pediatrics [38]	PubMed	2002-2007

18	Rationale use of medicine [39]	PubMed, Web of Science, Google Scholar, CINAHL, Proquest, International Pharmaceutical Abstract and Persian databases including SID, Iran Medex and MagIran	1975-2012
19	Neuroscience [40]	Science Citation Index Expanded (SCIE) via Web of Science database	2005-2008
20	Parasitology [41]	Web of Science	2002-2010
21	Stem cell [42]	Science Citation Index (SCI) Expanded	1996-2012
22	Stem cell [43]	Web of Science	2004-2010
23	Stem cell [44]	Web of Science	1996-2013
24	Health policy [45]	Web of Science	1898-2013
25	Cardiovascular diseases [46]	Medline	2003-2012
26	Obesity/overweight [47]	Scopus	1990-2013
27	Health impacts of mustard gas exposure in Iran-Iraq war [48]	Scopus, Medline, and Web of Science	1988-2012
28	Diabetes [49]	Scopus, Medline, and Web of Science	1990-2012
29	Diabetes [50]	Scopus	1968-2014
30	Reproductive health [51]	Scopus	2010-2014
31	Public health [52]	Web of Science and PubMed	1975-2014
32	Hepatitis [53]	Web of Science	2005-2014
33	Breast cancer [54]	Scopus	1991-2015
34	Neurosurgical research [55]	PubMed and IranMedex were searched for the publications of all Iranian neurosurgeons and also the neurosurgeons were invited to send the list of their publications	Before 1990; 1991-2000, and after 2000

References

1 Kolahi AA, Tahmooreszadeh S, Gachkar L, Mardani M. Research Priority Setting of Shaheed Beheshti Medical University Infectious Diseases and Tropical Medicine Research Center in 2007. Arch Clin Infect Dis. 2008;3.

2 Ghanbari A, Baghaiee M. Nurses' perspectives of nursing research priorities about cancer patients. IJN. 2009;22:87-97.

3 Kolahi A-A, Sohrabi M-R, Abdollahi M, Soori H. Setting research priority in Shahid Beheshti University of Medical Sciences: methods, challenges, limitations. Pajoohandeh Journal. 2010;15:143-51.

4 Farsar A-R, Khaleghnejad-Tabari A, Sadeghian N, Roozroukh M, Sohrabi M-R, Monfared AB, et al. Research priority setting for Pediatric Surgery Research Center of Shahid Beheshti University of Medical Sciences in 2010. Pajoohandeh Journal. 2013;18:139-47.

5 Abachizadeh K, Mohagheghi M-A, Mosavi-Jarrahi A. Setting research priorities to reduce burden of cancer in Iran: an institutional experience. Asian Pacific journal of cancer prevention : APJCP. 2011;12:2365-70.

6 Bahadori M, Ravangard R, Farzaneh A, Hakimzadeh SM. Research needs assessment in the health insurance organization: Level of health care provider. Electron Physician. 2011;3:436-41.

7 Sohrabi MR, Roodsari MR, Souri H, Mortazavi M, Ghanbari S, NazarAli S, et al. Research Priorities of Health System Research (HSR) in Vice-Chancellors of the Shahid Beheshti University of Medical Sciences. Journal of Isfahan Medical School. 2011;28.

8 Bahadori M, Teimourzadeh E, Farzaneh A, Nejati M. Prioritizing research needs: insights from a healthcare organization in Iran. Arch Pharm Pract. 2011;2:135.

9 Amini M, Kojuri J, Lotfi F, Karimian Z, Abadi A. Research priorities in medical education in the Eastern Mediterranean Region. East Mediterr Health J. 2012;18:687.

10 Tootoonchi M, Yamani N, Changiz T, Yousefy A. Research priorities in medical education: A national study. J Res Med Sci. 2012;17:83.

11 Bigdeli M, Javadi D, Hoebert J, Laing R, Ranson K. Health policy and systems research in access to medicines: a prioritized agenda for low-and middle-income countries. Health Res Policy Syst. 2013;11:37.

12 Ravaghi H, Sajadi HS. Research priorities for patient safety in Iran. J Patient Saf. 2015. DOI: 10.1097/PTS.0000000000000215 , PMID: 26076078 Issn Print: 1549-8417

13 Bahadori M, Izadi M, Karamali M, Teymourzadeh E, Yaghoubi M. Research priorities in a military health organization using multi criteria decision making techniques. Journal of military medicine. 2014;16:37-44.

14 Sohrabi M-R, Tahmoreszadeh S, Mohammadi F, Kolahi A-A. Research Priority Setting for Social Determinants of Health Research Center of Shahid Beheshti University of Medical Sciences in 2013. Community health. 2015;1:32-44.

15 Pourhosseini SS, Dehnavieh R, Mehrolhassani MH. Research Priorities of Kerman Medical Sciences University: A Documentary Study. Journal of the Society for Applied Socio-Economic Research and Development. 2015;3:293-303.

16 Nabeiei P, Amini M, Ghanavati S, Marhamati S. Research priorities in medical education at Shiraz University of Medical Sciences: categories and subcategories in the Iranian context. J Adv Med Educ Prof. 2016;4:26.

17 Majidi A, Ghiasvand R, Hadji M, Nahvijou A, Mousavi A-S, Pakgohar M, et al. Priority setting for improvement of cervical cancer prevention in Iran. Int J Health Policy Manag. 2016;5:225.

18 Doosti-Irani A, Holakouie-Naieni K. Determination the Research Priorities in the Field of HIV/AIDS in Iran: A Systematic Review Article. Iran J Public Health. 2016;45:1149.
19 Farshad AA, Mirkazemi R, Taheri F, Moridi P, Ghaemi Mood S, Alamdar M. Research priority in occupational health. Iran Occupational Health Journal. 2017;14:123-33.
20 Azizi F. Published researches in medical education in Iran. Iranian Journal of Medical Education. 2000;1:3-7.
21 Aslani J, Khedmat H, Assari S, Khoddami-Vishte HR, Alaeddini F, Einollahi B, et al. Transplantation research in Iran: a bibliometric study. Transplantation proceedings. 2007;39:788-9.
22 Einollahi B. Nephrology research output in Iran in a decade. Iranian journal of kidney diseases. 2007;1:57-62.
23 Einollahi B, Motalebi M, Taghipour M, Ebrahimi M. Iranian Nephrology and Urology Research Output in the Past Two Decades: A Bibliographic Analysis of Medline Database. Nephrourol Mon. 2015;7:e30911.
24 Farhoudian A, Goodarzi RR, Movaghar AR, Sharifi V, Mohammadi MR, Izadian ES, et al. Trend of researches in the field of psychiatric disorders in Iran. Iranian Journal of Psychiatry and Clinical Psychology. 2007;12:327-36.
25 Sharifi V, Rahimi-Movaghar A, Mohammadi MR, Goodarzi RR, Izadian ES, Farhoudian A, et al. Analysis of mental health research in the Islamic Republic of Iran over 3 decades: a scientometric study. East Mediterr Health J. 2008;14:1060-9.
26 Rahimi-Movaghar A, Amin-Esmaeili M, Safarcherati A, Sarami H, Rafiey H. A Scientometric Study of Iranian Scientific Productions in the Field of Substance Use and Addiction Research in the Years 2008 to 2012. Addiction & health. 2015;7:99-108.
27 Rasolabadi M, Rasouli-Ghahfarkhi SM, Ardalan M, Kalhor MM, Seidi J, Gharib A. Epilepsy Research in Iran: a Scientometric Analysis of Publications Output During 2000-2014. Acta Inform Med. 2015;23:374-8.
28 Assari S, Ahmadyar M. Dental research in Iran: a bibliometric analysis of electronically available literature. Int Dent J. 2009;59:210-4.
29 Sadeghi M, Shahravan A, Haghdoost AA, Asgary S, Rad M. Trend in dental research output in Iran over a period of 20 years (1990-2009). Int Dent J. 2012;62:84-9.
30 Yaminfirooz M, Motallebnejad M, Gholinia H, Esbakian S. Quantitative and Qualitative Evaluation of Iranian Researchers' Scientific Production in Dentistry Subfields. Acta Inform Med. 2015;23:301-5.
31 Asgary S, Sabbagh S, Shirazi AS, Ahmadyar M, Shahravan A, Akhoundi MS. PubMed-Indexed Dental Publications from Iran: A Scientometric Study. J Dent (Tehran). 2016;13:157-67.
32 Asgary S, Sabbagh S, Eghbal MJ. Published endodontic articles in PubMed-indexed journals from Iran. Iran Endod J. 2012;7:1.
33 Eghbal MJ, Davari Ardakani N, Asgary S. A Scientometric Study of PubMed-Indexed Endodontic Articles: A Comparison between Iran and Other Regional Countries. Iran Endod J. 2012;7:56-9.
34 Badri S, Akhoundi MS, Fard MJ, Momeni N, Hedayati Z, Vakili V. Orthodontic research output from iran in international and national journals. J Dent (Tehran). 2014;11:67-74.
35 Asgary S, Motazedian HR, Parirokh M, Eghbal MJ, Kheirieh S. Twenty years of research on mineral trioxide aggregate: a scientometric report. Iran Endod J. 2013;8:1-5.
36 Khaji A, Ghodsi SM, Eftekhar B, Karbakhsh M. Trauma research in Iran: a report of the Sina Trauma Data Bank. Arch Iran Med. 2010;13:17-20.
37 Katibeh M, Moein H-R, Javadi M-A. Contribution of Iran to the ophthalmic literature over the past three decades. J Ophthalmic Vis Res. 2011;6:225.

38 Malekahmadi MR, Moazen B, Khoshdel A, Rahimzadeh F, Lankarani MM. Iranian research output in pediatrics: 1975-2007. J Res Med Sci. 2011;16:1605-11.
39 Mousavi S, Mansouri A, Ahmadvand A. A bibliometric study of publication patterns in rational use of medicines in Iran. Pharm Pract (Granada). 2013;11:38-43.
40 Ashrafi F, Mohammadhassanzadeh H, Shokraneh F, Valinejadi A, Johari K, Saemi N, et al. Iranians' contribution to world literature on neuroscience. Health Information & Libraries Journal. 2012;29:323-32.
41 Rashidi A, Rahimi B, Delirrad M. Bibliometric analysis of parasitological research in iran and Turkey: a comparative study. Iran J Parasitol. 2013;8:313-22.
42 Ahmadi M, Habibi S, Sedghi S, Hosseini F. Bibliometric analysis of stem cell publications in iran. Acta Inform Med. 2014;22:259-62.
43 Gheisari Y, Baharvand H, Nayernia K, Vasei M. Stem cell and tissue engineering research in the Islamic Republic of Iran. Stem Cell Rev. 2012;8:629-39.
44 Alijani R. Rapid Scientific Promotion of Scientific Productions in Stem Cells According to The Indexed Papers in The ISI (web of knowledge). Cell J. 2015;17:372-8.
45 Kalankesh LR, Kalankesh LR. Publication on health policy worldwide from 1898 to 2013: identifying position of iran. Mater Sociomed. 2014;26:419-21.
46 Al-Kindi S, Al-Juhaishi T, Haddad F, Taheri S, Abi Khalil C. Cardiovascular disease research activity in the Middle East: a bibliometric analysis. Ther Adv Cardiovasc Dis. 2015;9:70-6.
47 Djalalinia S, Peykari N, Qorbani M, Moghaddam SS, Larijani B, Farzadfar F. Obesity Researches Over the Past 24 years: A Scientometrics Study in Middle East Countries. Int J Prev Med. 2015;6:38.
48 Nokhodian Z, ZareFarashbandi F, Shoaei P. Mustard gas exposure in Iran-Iraq war - A scientometric study. J Educ Health Promot. 2015;4:56.
49 Peykari N, Djalalinia S, Kasaeian A, Naderimagham S, Hasannia T, Larijani B, et al. Diabetes research in Middle East countries; a scientometrics study from 1990 to 2012. J Res Med Sci. 2015;20:253.
50 Rasolabadi M, Khaledi S, Ardalan M, Kalhor MM, Penjvini S, Gharib A. Diabetes research in Iran: a scientometric analysis of publications output. Acta Inform Med. 2015;23:160.
51 Bazm S, Kalantar SM, Mirzaei M. Bibliometric mapping and clustering analysis of Iranian papers on reproductive medicine in Scopus database (2010-2014). Int J Reprod Biomed (Yazd). 2016;14:371-82.
52 Poreau B. Scientometrics on Public Health Research in Iran: Increase of Area Studies despite Embargoes? A Review Article. Iran J Public Health. 2017;46:281-5.
53 Rezaee Zavareh MS, Alavian SM. Ten-year analysis of hepatitis-related papers in the Middle East: a web of science-based scientometric study. Turk J Gastroenterol. 2017;28:20-5.
54 Zarei M, Bagheri-Saweh MI, Rasolabadi M, Vakili R, Seidi J, Kalhor MM, et al. Breast cancer research in Iran: a scientometric analysis of publications output from 1991 to 2015 in Scopus. Electron physician. 2017;9:3816-22.

55 Alimi M, Taslimi S, Ghodsi SM, Rahimi-Movaghar V. Quality and quantity of research publications by Iranian neurosurgeons: Signs of scientific progress over the past decades. Surg Neurol Int. 2013;4:38.
